# Sustainable chromatographic assays of a novel antifungal combination for keratomycosis

**DOI:** 10.1186/s13065-026-01735-y

**Published:** 2026-03-16

**Authors:** Faten M. Aboukhalil, Essam F. Khamis, Mahmoud A. El-Sayed, Amira F. El-Yazbi, Rasha M. Youssef

**Affiliations:** Pharmaceutical Analytical Chemistry Department, Faculty of Pharmacy, Alexandria University, Alexandria, Egypt

**Keywords:** Keratomycosis, Fluconazole, Natamycin, RP-HPLC, Capillary zone electrophoresis, VIGI, Greenness, Whiteness and Blueness approaches

## Abstract

Keratomycosis, a sight-threatening fungal infection of the cornea, can be more effectively treated with a novel combination of Natamycin (NAT, 5%) and Fluconazole (FLC, 0.2%) than with NAT alone. To support the development of this therapy, new sustainable, eco-friendly, and sensitive analytical methods were developed for the simultaneous quantification of both drugs in pharmaceutical formulations. Two techniques were successfully employed. The first was a Reversed-Phase High-Performance Liquid Chromatography (RP-HPLC) method using an isocratic mobile phase of methanol and water (70:30%v/v) at a 1 mL/min flow rate. Detection was accomplished with both diode array (DAD) and fluorescence (FLD) detectors. The second technique was a Capillary Zone Electrophoresis (CZE) method utilizing a capillary (56 cm effective length, 50 μm internal diameter) at ambient temperature. Samples were injected hydrodynamically at 50 mbar pressure. The environmental sustainability and practical applicability of these methods were rigorously evaluated using modern assessment tools: the Greenness, Whiteness, and Blueness approaches. Furthermore, their innovative character was formally quantified using a novel metric, the Violet Innovation Grade Index (VIGI).

**Supplementary Information:**

The online version contains supplementary material available at 10.1186/s13065-026-01735-y.

## Introduction

**Fig. 1:**
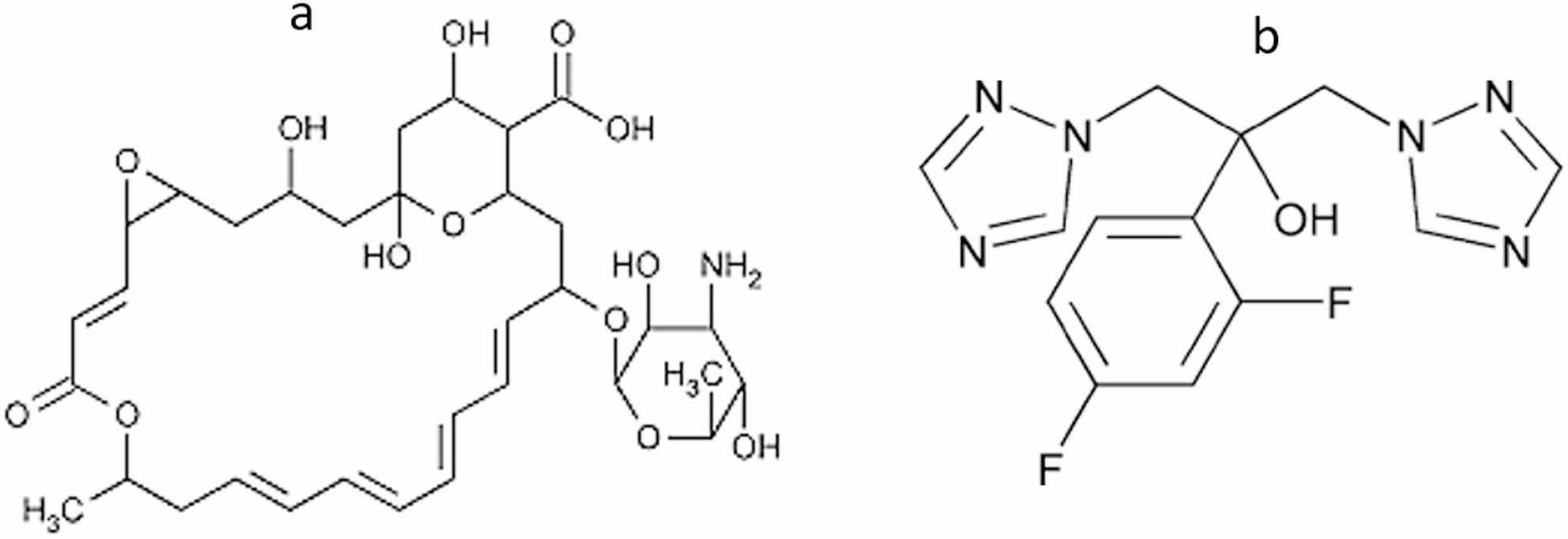
Chemical structure for **a** natamycin and **b** fluconazole

Natamycin (NAT) ((1R,3 S,5R,7R,8E,12R,14E,16E,18E,20E,22R,24 S,25R,26 S)-22-[(3-amino-3,6-dideoxy-β-D-mannopyranosyl)oxy]-1,3,26-trihydroxy-12-methyl-10-oxo-6,11,28-trioxatricyclo [22.3.1.05,7]octacosa-8,14,16,18,20-pentaene-25-carboxylic acid) [1] which is used as food preservative and as antifungal medication is a natural antimycotic agent (Fig. 1a). It is effective in preserving fermented foods and also used in ophthalmic medications to treat fungal infections [2]. It is found that NAT is applicable for treatment of conjunctivitis, blepharitis and keratitis [3]. Fluconazole (FLC) ( 2-(2,4-difluorophenyl)-1,3-bis(1,2,4-triazol-1-yl)propan-2-ol) [4] is an antifungal drug used for both superficial and systemic fungal infections (Fig. 1b). FLC has a powerful advantage over other antifungal drugs like the possibility of oral administration with minimal side effects [5]. For treatment of fungal keratitis, combined therapy of 5% NAT and 0.2% FLC has more efficacy than monotherapy of NAT alone. This occurs due to the force of penetration of FLC into deep corneal tissues but NAT is effective only at superficial layer of cornea [6].

The literature reviews lack any analytical methods for simultaneous determination of NAT and FLC in this ophthalmic combination. The challenge of the proposed study is to provide, for the first time, new and unique sustainable chromatographic methods to overcome the high concentration ratio of NAT and FLC (25:1) in dosage form. The proposed HPLC work aims to develop rapid, sensitive and green technique for simultaneous determination of NAT and FLC in topical dosage form. Also, NAT alone can be determined by fluorescence detector (FLD) at λ_ex_ = 305 nm and λ_em_ = 404 nm which provides sensitive method for determination of NAT in pharmaceutical preparation. Moreover, Capillary zone electrophoresis (CZE) offers variable advantages such as rapid time of analysis, eco-friendly, high resolution, minimal sample consumption and minimal waste production. Thus, the proposed HPLC method is recommended for stability-indicating and high-sensitivity assays, while the CZE method is an excellent, eco-friendly alternative for rapid routine analysis and as a confirmatory technique.

Our work directly supports the advancement of this promising combination therapy. The cited literature [6] demonstrates that the 5% NAT / 0.2% FLC combination has superior efficacy precisely due to their complementary mechanisms and pharmacokinetics. However, inaccurate dosing could compromise the well-documented synergistic efficacy [6]. Sub-therapeutic levels of NAT might fail to eradicate superficial fungi, while inadequate FLC could permit deep-tissue invasion. Therefore, the proposed analytical methods serve as essential quality control tool to ensure that patients receive the precise, potent formulation demonstrated to be effective in clinical settings. The method presented here is the first capable of simultaneously quantifying this specific ratio in a dosage form. It therefore provides the essential analytical backbone for manufacturing quality assurance, ensuring that the final product delivered to patients possesses the exact chemical composition that underpins its clinical advantage over monotherapy. Moreover, it is acknowledged that NAT is susceptible to degradation under various stress conditions [7–10]. The methods presented here provide a foundational analytical tool for future, dedicated stability-indicating studies of this specific combination, which are planned to comprehensively profile its degradation behavior. The high sensitivity, particularly of the HPLC-FLD method for NAT, is well-suited for such subsequent investigations into trace-level degradants.

Sustainable analytical chemistry or White Analytical Chemistry (WAC) is the development of analytical methodologies that align with environmental, economic, and societal sustainability while maintaining scientific reliability. It is increasingly recognized as a critical discipline in advancing global strategies for environmental pollution mitigation. The concept of WAC has been established in the literature for over five years. It presents a new perspective for implementation of Sustainable Development (SD) principles in analytical chemistry, inspired by the RGB model. A persistent challenge within the discipline of sustainable analytical chemistry is the rigorous assessment of the greenness, whiteness & blueness of analytical methodologies. It is a well-established principle in scientific inquiry and process management that what cannot be quantitatively assessed cannot be systematically regulated, improved, or validated. Sustainable Analytical Chemistry has been discussed in many literatures. These literatures [11–19] established several new tools for assessment of greenness and whiteness approaches.

In light of this, the present investigation is directed toward the rational design, methodological development, and comprehensive optimization of analytical strategies for simultaneous determination of NAT and FLC by two proposed chromatographic methods; RP-HPLC and CZE. Several greenness assessment tools were established for comparing the two proposed methods including: National environmental method index (NEMI) [11], modified NEMI [12], analytical eco-scale [13, 14], AGREE tool [15] and green analytical procedure index (GAPI) [14, 16]. HPLC environmental assessment (HPLC-EAT) was applied to evaluate the sustainability of RP-HPLC method [17]. Likewise, Red-Green-Blue method (RGB) which contains twelve principles is an extension of the twelve principles of Green Analytical Chemistry (GAC) [18, 19]. RGB is a crucial approach to guarantee that the analytical procedures are efficient and economic. Also, Blue applicability grade index (BAGI) is a new tool for ensuring method practicality. BAGI is a complementary tool to GAC which involves number of samples, reagents types, sample preparation, instrument type and number of simultaneously treated samples [20, 21].

Also, Violet Innovation Grade Index (VIGI) is a new, valuable and comprehensive approach in analytical field for evaluation of innovation of the analytical procedures. VIGI involves ten different criteria as preparation of sample and instrumentation, data processing and software, WAC and its derivatives, sensitivity of the analytical method, automation grade, materials and reagents, miniaturization, interdisciplinarity and approach providing. VIGI is represented as decagon star shaped pictogram with three color scale, dark purple, light purple and white which indicates high, moderate and low innovation level of analytical method, respectively. Likewise, the degree of innovation can be obtained from score in the center of the pictogram. Score value of 50 or more ensures the high innovation of the analytical method [22].

## Experimental

### Instrumentation

#### HPLC method

The HPLC Agilent 1200 series formed of quaternary pump, auto-injector, vacuum degasser; Diode Array Detector G1315 C/D, multiple wavelength detector G1365 C/D. The used software was Agilent ChemStation (Agilent Technologies, Santa Clara, USA). The separation was performed using Agilent Zorbax Eclipse plus C18 analytical column with the following dimensions 4.6 × 250 mm, 5 Micron.

#### CZE method

Agilent CE instrument 7100 series (Agilent Technologies Deutschland, GmbH, Hewlett-Packard-Str.8 Waldbronn, Germany) equipped with a Diode Array Detector was used. The measurements were performed on a deactivated fused silica capillary obtained from Agilent Technologies (Waldbronn, Germany). It had the following dimensions: 56 cm effective length and 50 μm id. Measurements were performed at ambient temperature. Samples were injected with hydrodynamic mode by applying pressure equal to 50 mbar. The data were recorded and processed by Agilent ChemStation Software. PH meter JENWAY (model 3505) was used for adjustment of pH of the solutions.

### Materials

NAT and benzalkonium chloride were gift from Orchidia Pharmaceutical Industries, Al-Obour city- Industrial zone, Area 15, Block no. 12,011, Cairo, Egypt. FLC was purchased from Pfizer Company, Giza, Egypt. Natamycin^®^ 5% eyedrop and Diflucan^®^ intravenous solution were obtained from Egyptian markets. Boric acid and ortho-Phosphoric acid 85% were obtained from El-Nasr Chemical Industry Company. Sodium hydroxide was purchased from Union Drug & Chemical Company. HPLC grade methanol was a product of Sigma- Aldrich Company.

### HPLC method

#### Chromatographic conditions

The applied separation system performed depending on isocratic system at 1 mL/min flow rate. The mobile phase is HPLC grade methanol (CH_3_OH): distilled water in ratio 70:30%v/v. Filtration of mobile phase is performed using 0.45 μm membrane filter. The separation was applied at room temperature (25 °C) with 10 µL injection volume. DAD detection system ensures high sensitivity by determining each drug at its maximum wavelength where NAT was determined at 270 nm and FLC at 210 nm. Also, DAD detector has another advantage as it ensures the purity of the drug and absence of any interference by providing peak purity plots. Likewise, NAT can be determined using FLD with higher sensitivity than DAD.

### Capillary zone electrophoresis method

First of all, the capillary was flushed with 0.5 M NaOH for 15 min followed by flushing with water for 15 min. The next step was flushing with 0.1 M NaOH for 10 min. Application of waiting step for 5 min was so important step to ensure activation of the inner silica wall of the capillary. Then, the capillary is washed with water for 10 min. Finally, flushing of running buffer of borate pH 10 is applied for 10 min.

The optimal conditions to obtain the best baseline, sharp peaks and good resolution were achieved by using 100 mM borate buffer pH 10 with 50 mbar applied pressure as hydrodynamic mode for 3 s injection time at applied voltage equal to 30 kV. Evaluation of the peak purity was performed by using Agilent ChemStation Software. The applied wavelengths ensured pure separation of each drug either in bulk powder or in pharmaceutical preparation where the purity ratio appeared on the green region not on red one which indicated purity of each peak.

### Preparation of stock, working solutions and synthetic mixtures

2 mg/mL stock solution of each drug and 1 mg/mL stock solution of BNZ were prepared in 10-mL volumetric flask separately in HPLC grade methanol. Then 200 µg/mL solution of BNZ was prepared in methanol from its stock solution. Working solutions were prepared by transferring different volumes from stock solutions into 10 mL-volumetric flaks and dilution was made to volume with distilled water to obtain 5–250 µg/mL and 10–200 µg/mL in RP-HPLC and 100–1250 µg/mL and 30–250 µg/mL in CZE for NAT and FLC, respectively. Synthetic mixtures were prepared by dilution of working solutions in distilled water by different ratios to get different concentrations through linearity ranges.

### Analysis of laboratory-prepared ophthalmic solution

Laboratory-prepared solution (LPS) was made to contain 5% NAT, 0.2% FLC and 0.02% benzalkonium chloride (BNZ) as preservative. After step dilution of LPS, working solutions were prepared in distilled water to contain 250 µg/mL NAT: 10 µg/mL FLC: 1 µg/mL BNZ and 1000 NAT µg/mL: 40 µg/mL FLC: 4 µg/mL BNZ for analysis using RP-HPLC and CZE methods, respectively. Triplicate determinations were performed for each solution.

### Analysis of pharmaceutical preparations

Stock solutions of 1000 µg/mL were prepared separately by reconstitution of Natamycin^®^ 5% eyedrop and Diflucan^®^ intravenous solution in water. Working solutions were prepared separately in distilled water. Each solution was measured three times following the proposed methods to study reproducibility and repeatability.

## Results and discussion

It is valuable to mention that it is the first time to simultaneously analyze our investigated drugs. There are no any analytical literature reviews reported for simultaneous analysis of NAT and FLC.

### Method development and optimization of HPLC method

For accurate, sensitive analysis of the binary mixture, chromatographic conditions should be examined to reach optimum conditions. We studied different parameters that affected the investigated drugs separation and their effect on system suitability parameters (SSP). The studied parameters involved mobile phase composition, mobile phase ratio and injection volume.

#### Mobile phase composition

Different buffers with different pHs and distilled water were studied with organic modifiers to obtain the best peak shape with good resolution. Buffers with pH 3, 5, 7, 8 and 9 were studied using different buffer types including phosphate, acetate and borate buffer. It was found that the peak shapes were distorted at acidic pHs but NAT peak disappeared at alkaline pHs. The best separation of the peaks and accepted sharp peaks occurred with water and organic modifier (methanol).

#### Mobile phase ratio

**Fig. 2:**
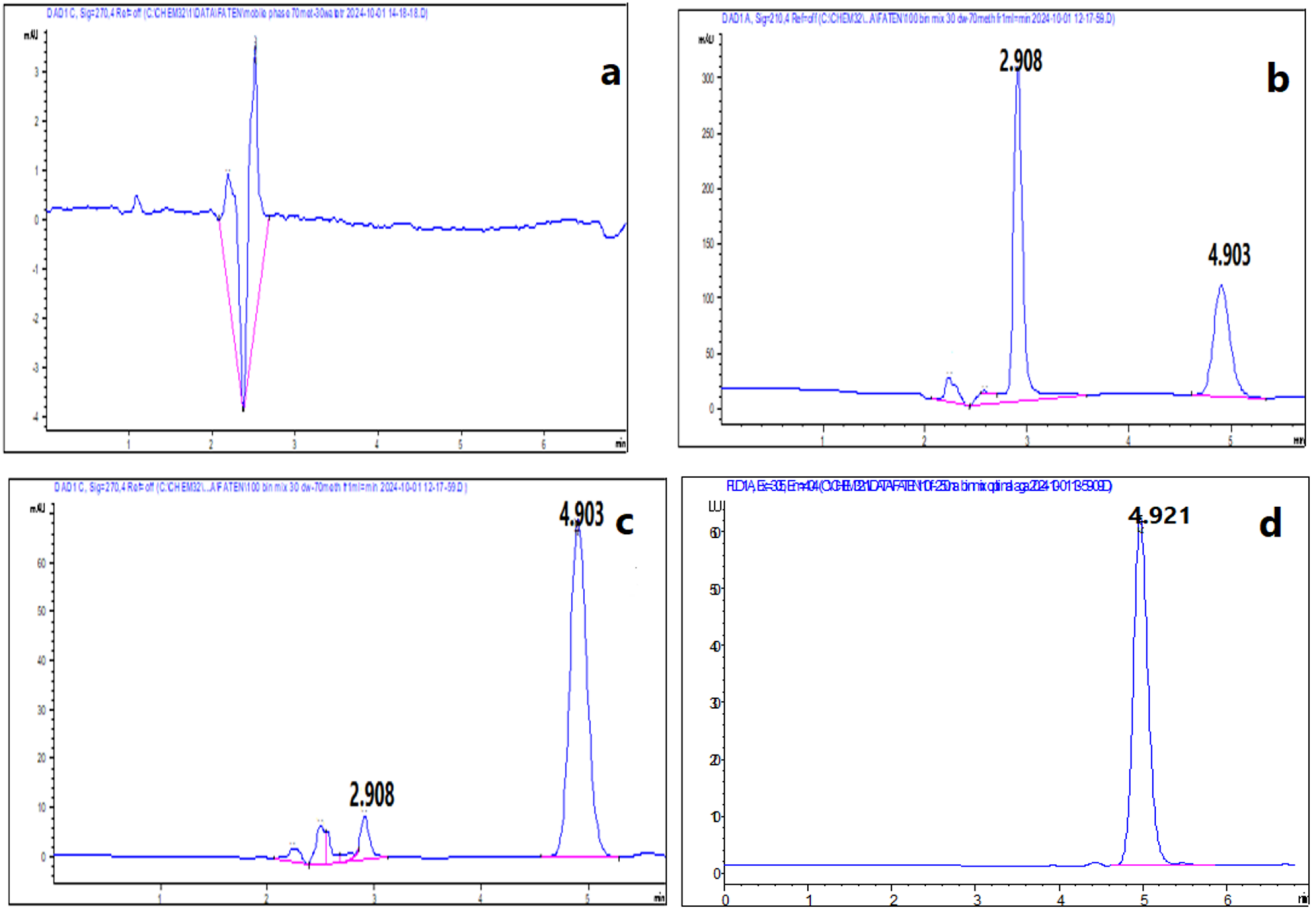
Typical HPLC chromatograms of **a** blank, standard synthetic mixture containing 100 µg/mL of NAT at 4.9 min and 100 µg/mL FLC at 2.9 min **b** using DAD at 210 nm, **c** using DAD at 270 nm and **d** using FLD at λ_ex_ = 305 nm and λ_em_ = 404 nm

Different ratios of methanol to water were examined. The result was that water ratio more than 35% gave long separation time where NAT appeared at separation time longer than 10 min. The optimal separation time was obtained at methanol to water ratio 70:30%v/v where FLC and NAT were separated at 2.9 and 4.9 min, respectively (Fig. 2).

#### Injection volume

Studying different injection volumes from 5 µL to 20 µL to obtain high sensitivity with accepted peak shape led to the selection of 10 µL injection volume.

### Optimization of capillary zone electrophoresis

To obtain optimal separation conditions, several parameters such as buffer concentration, buffer pH, injection time and voltage were studied for simultaneous analysis of the binary mixture of NAT and FLC. The aim of optimization procedure is to achieve sharp peaks and better baseline with accepted SSP.

#### Buffer pH

Different buffer types with different pHs were examined (from pH 3 to pH 11). It was observed that NAT peak disappeared at acidic pHs but began to appear starting from pH 7 with bad sensitivity of FLC peak. Good sensitivity and resolution were achieved with borate buffer pH 10.

#### Buffer concentration

**Fig. 3:**
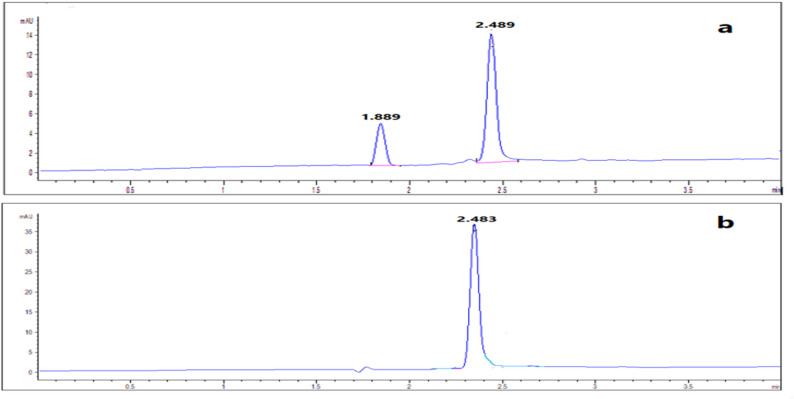
Typical electropherograms of a synthetic mixture containing 500 µg/mL NAT and 80 µg/mL FLC **a** using DAD at 210 nm and **b** using DAD at 305 nm by using 100 mM borate buffer, pH 10 at 30 kV applied voltage using hydrodynamic injection at 50 mbar for 3 s

Several concentrations of borate buffer were studied ranging from 10 mM to 200 mM. It was found that concentrations below 100 mM produced too short separation time while those more than 100 mM produced distorted peaks with bad baseline. So, the optimum buffer concentration was 100 mM borate buffer which separated FLC at 1.89 min and NAT at 2.48 min (Fig. 3).

#### Injection time

Injection times from 3 s to 30 s were studied. A 3-sec injection time at 50 mbar applied pressure produced sharp peaks of the investigated drugs with achieving a good linearity. Increasing injection time led to production of broad peaks.

#### Applied voltage

Different applied voltages from 15 kV to 30 kV were tested with 5-kV intervals. It was observed that all measurements with applied voltage below 30 kV were time consuming with long migration time. A 30-kV applied voltage achieved the accepted capacity factor.

### Detection wavelength

**Fig. 4:**
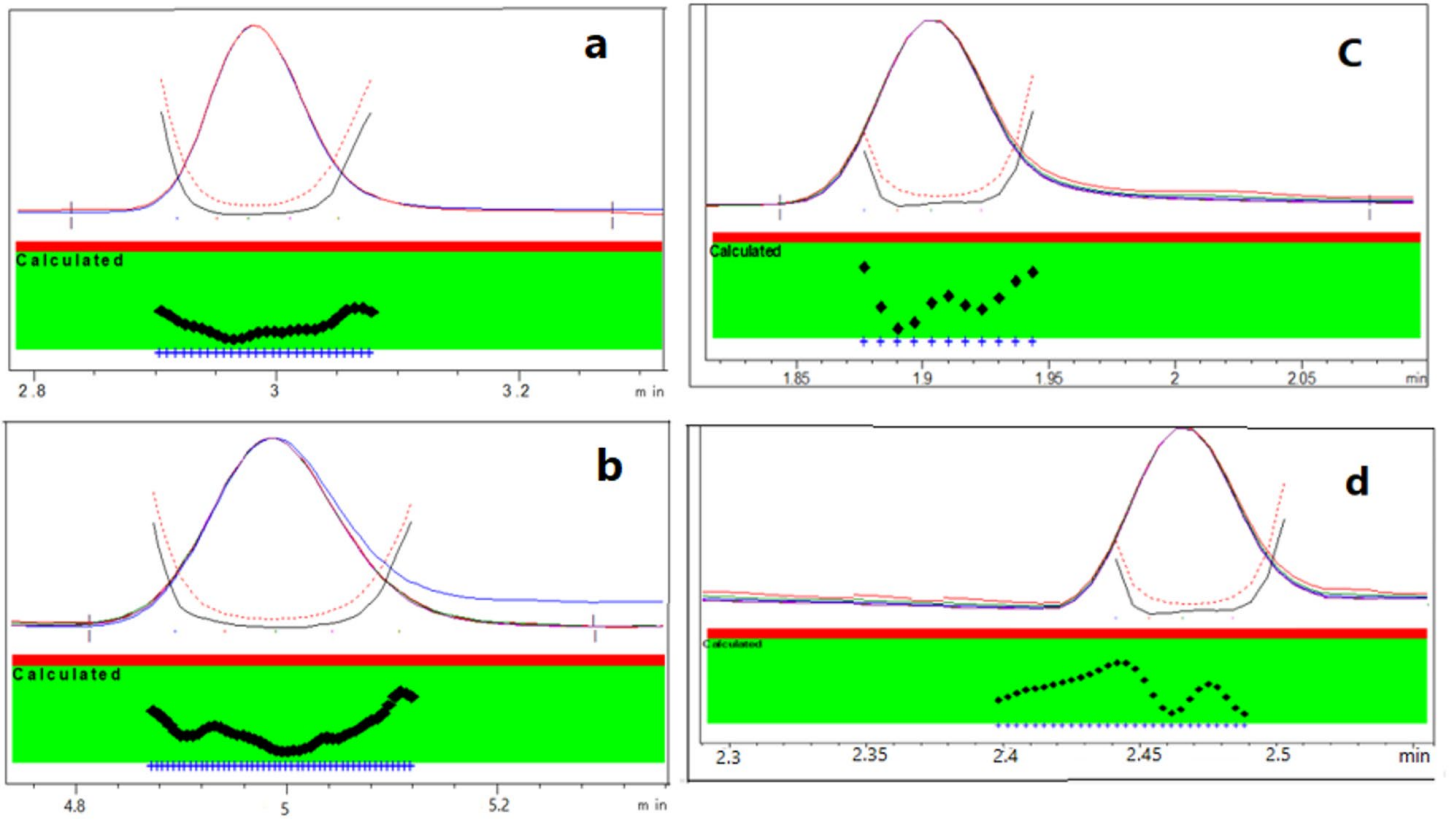
Peak purity plots **a** and **b** using RP-HPLC, while **c** and **d** using CZE for FLC and NAT, respectively

**Fig. 5:**
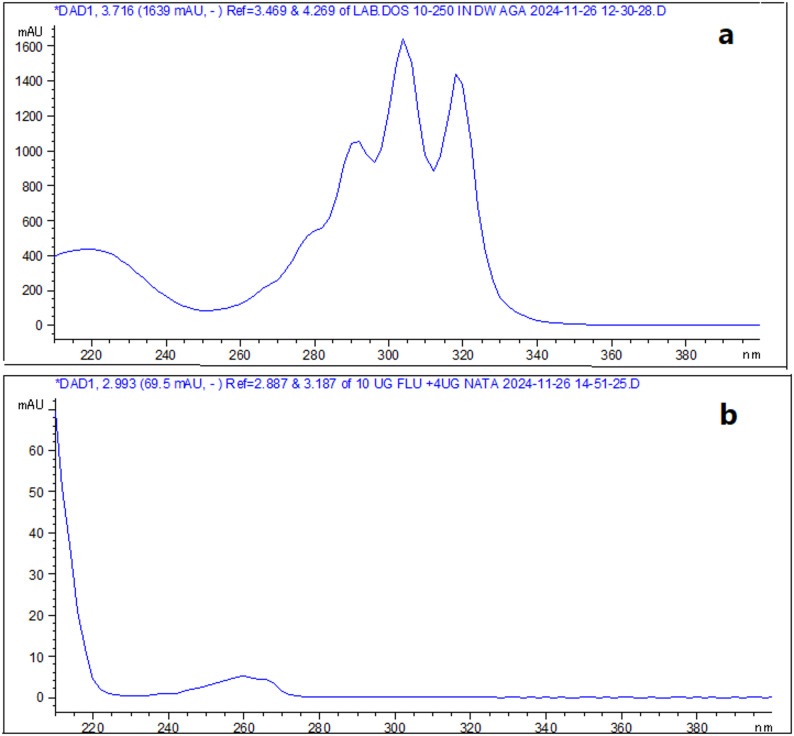
UV spectra of **a** NAT and **b** FLC

DAD provided many advantages such as measuring of investigated drugs at different wavelengths at the same time, selection maximum wavelength for each drug to provide higher sensitivity and selectivity and providing peak purity plots for each drug to ensure analysis of investigated drugs without any interference as shown in Fig. 4 for the proposed methods. So, NAT and FLC were measured using RP-HPLC at 270 nm and 210 nm and using CZE at 305 nm and 210 nm, respectively. Also, DAD has the ability to provide UV spectrum of each of investigated drugs (Fig. 5). FLD has been used to detect NAT at λ_ex_ = 305 nm and λ_em_ = 404 nm as FLD provides higher sensitivity and selectivity than DAD with better signal to noise. The LOD for NAT using DAD was 1.53 µg/mL, whereas with FLD it was dramatically improved to 0.03 µg/mL (a ~ 50-fold increase in sensitivity). This ultra-sensitive FLD method is crucial for detecting trace-level degradation products or for potential future applications in bioavailability studies where NAT concentrations are very low.

### System suitability parameters (SSP)

SSP is a valuable tool to ensure the effectiveness and suitability of the selected chromatographic system to obtain accepted separation time, good resolution, sharp peak shape and high efficiency of separation. Theses parameters were studied for the two proposed methods (Table 1).

**Table 1:** System suitability parameters for the analysis of binary mixture of FLC and NAT using HPLC and CZE methods

Parameters	HPLC method			CZE method	
	FLC	NAT (DAD)	NAT (FLD)	FLC	NAT
t_R_ ± SD (min)^a^	2.91 ± 0.02	4.90 ± 0.03	4.92 ± 0.02	1.89 ± 0.03	2.49 ± 0.04
Retention factors (k’)	2.23	5.125	5.05	2.77	2.98
Theoretical plate (N)	7869	3567	4514	6780	5975
USP tailing factor(T)	1.20	1.40	1.30	1.20	1.30
Resolution (Rs)	6.43			2.45	
Selectivity factor (ɑ)	2.29			1.08	

^a^ Average of five determinations

- System suitability recommendations: k’> 2, R_s_ > 2, *N* > 2000, and T ≤ 2

### Validation

Simultaneous analysis of NAT and FLC has been validated using different parameters of validation according to International Conference on Harmonization (ICH) [23]. Linearity was examined by study the relationship between peak area and concentration either in HPLC method or CZE one for NAT and FLC which indicate high correlation coefficient (0.999 or more) as shown in Table 2. Accuracy and precision represented as %Er and %RSD, respectively have been performed by triplicate measurement of different concentrations of investigated drugs within inter-day analysis (Table 3). Precision was performed within inter-day and intra-day precision. Limit of detection (LOD) and limit of quantification (LOQ) determined according to signal to noice ratio (S/N) where LOD equals 3.3 S/N and LOQ equals 10 S/N. For studying robustness, the same procedure was followed with different flow rate and wavelengths for RP- HPLC method and with different pHs and concentrations of the buffer, different λ_max_ and injection volumes for CZE method (Table 4).

**Table 2:** Regression and statistical parameters for the determination of FLC and NAT using the proposed methods

Parameters	RP-HPLC Method			CZE Method	
	FLC at 210 nm	NAT (DAD) at 270 nm	NAT (FLD)	FLC at 210 nm	NAT at 305 nm
Linearity range (µg/mL)	10.0-200.0	5.0-250.0	1.0-250.0	30.0-250.0	100.0-1250.0
LOQ (µg/mL)	4.55	4.63	0.09	12.20	68.47
LOD (µg/mL)	1.50	1.53	0.03	4.03	22.59
Intercept (a)	215.47	-7.51	-2.40	2.86 × 10^− 1^	5.57
Slope (b)	15.86	9.01	2.94	2.19 × 10^− 1^	1.59 × 10^− 1^
Correlation coefficient (r)	0.9999	0.9999	0.9999	0.9999	0.9998
S_a_	7.21	4.17	1.22	2.12 × 10^− 1^	7.04 × 10^− 1^
S_b_	6.94 × 10^− 2^	3.39 × 10^− 2^	1.16 × 10^− 2^	1.39 × 10^− 3^	1.14 × 10^− 3^
S_b_^2^	4.82 × 10^− 3^	1.15 × 10^− 3^	1.34 × 10^− 4^	1.94 × 10^− 6^	1.29 × 10^− 6^
S_y/x_	12.47	8.23	3.12	2.68 × 10^− 1^	1.09
*F*	52179.88	70649.01	64509.83	24845.15	19539.99
Significance F	3.05 × 10^− 11^	1.91 × 10^− 13^	1.16 × 10^− 18^	9.72 × 10^− 9^	9.04 × 10^− 12^

**Table 3:** Intra-day and inter-day precision and accuracy for the analysis of FLC and NAT mixtures using the proposed methods

Concentration (µg/mL)		Proposed Method	Mean % Recovery ± SD (%)		%RSD			%E_*r*_	
FLC	NAT		FLC	NAT	FLC	NAT		FLC	NAT
**(a) Accuracy and intra-day precision**									
20	20	HPLC Method	99.90 ± 0.79	101.06 ± 1.38	0.79		1.38	-0.1	1.06
100	200		100.55 ± 0.63	101.01 ± 0.85	0.63		0.84	0.55	1.01
200	100		99.64 ± 0.27	99.40 ± 0.85	0.27		0.85	-0.36	-0.60
10	250		99.65 ± 1.34	99.74 ± 0.44	1.34		0.44	-0.35	-0.26
30	1250	CZE Method	100.39 ± 1.52	101.11 ± 0.55	1.51		0.54	0.39	1.11
100	100		99.49 ± 0.69	101.65 ± 1.58	0.69		1.55	-0.51	1.65
250	500		99.93 ± 0.39	98.27 ± 0.77	0.39		0.78	-0.07	-1.73
200	100		100.33 ± 0.80	99.55 ± 0.63	0.80		0.63	0.33	-0.45
**(b) Accuracy and inter-day precision**									
20	20	HPLC Method	99.90 ± 0.79	101.99 ± 0.64	0.79		0.63	-0.1	1.99
100	200		100.13 ± 0.37	101.75 ± 0.32	0.37		0.31	0.13	1.75
200	100		99.30 ± 0.22	99.32 ± 0.83	0.22		0.83	-0.70	-0.68
10	250		101.22 ± 0.89	99.45 ± 0.26	0.88		0.26	1.22	-0.55
30	1250	CZE Method	99.38 ± 0.88	100.97 ± 0.32	0.88		0.32	-0.62	0.97
100	100		100.09 ± 0.26	101.64 ± 1.58	0.26		1.55	0.09	1.64
250	500		99.93 ± 0.39	99.49 ± 0.73	0.39		0.73	-0.07	-0.51
200	100		100.33 ± 0.16	99.55 ± 1.09	0.16		1.09	0.33	-0.45

**Table 4:** Robustness evaluation for simultaneous determination of FLC and NAT using the proposed methods

Proposed method	Parameters	Average %recovery ± %RSD	
		**FLC**	NAT
**RP-HPLC**	**Flow rate (1 mL/min ± 0.1)**	101.57 ± 0.45	100.96 ± 1.31
	**Detection wavelength ± 2 nm**	101.13 ± 0.9	100.16 ± 0.96
**CZE**	**Buffer pH (10 ± 0.05)**	100.57 ± 0.85	98.33 ± 0.65
	**Buffer concentration (100 mM ± 0.1)**	101.13 ± 1.67	100.97 ± 0.98
	**Detection wavelength ± 2 nm**	99.27 ± 1.47	99.61 ± 0.66
	**Flow rate (1 mL/min ± 0.1)**	101.12 ± 1.67	100.57 ± 1.11

### Analysis of laboratory-prepared ophthalmic solution

**Fig. 6:**
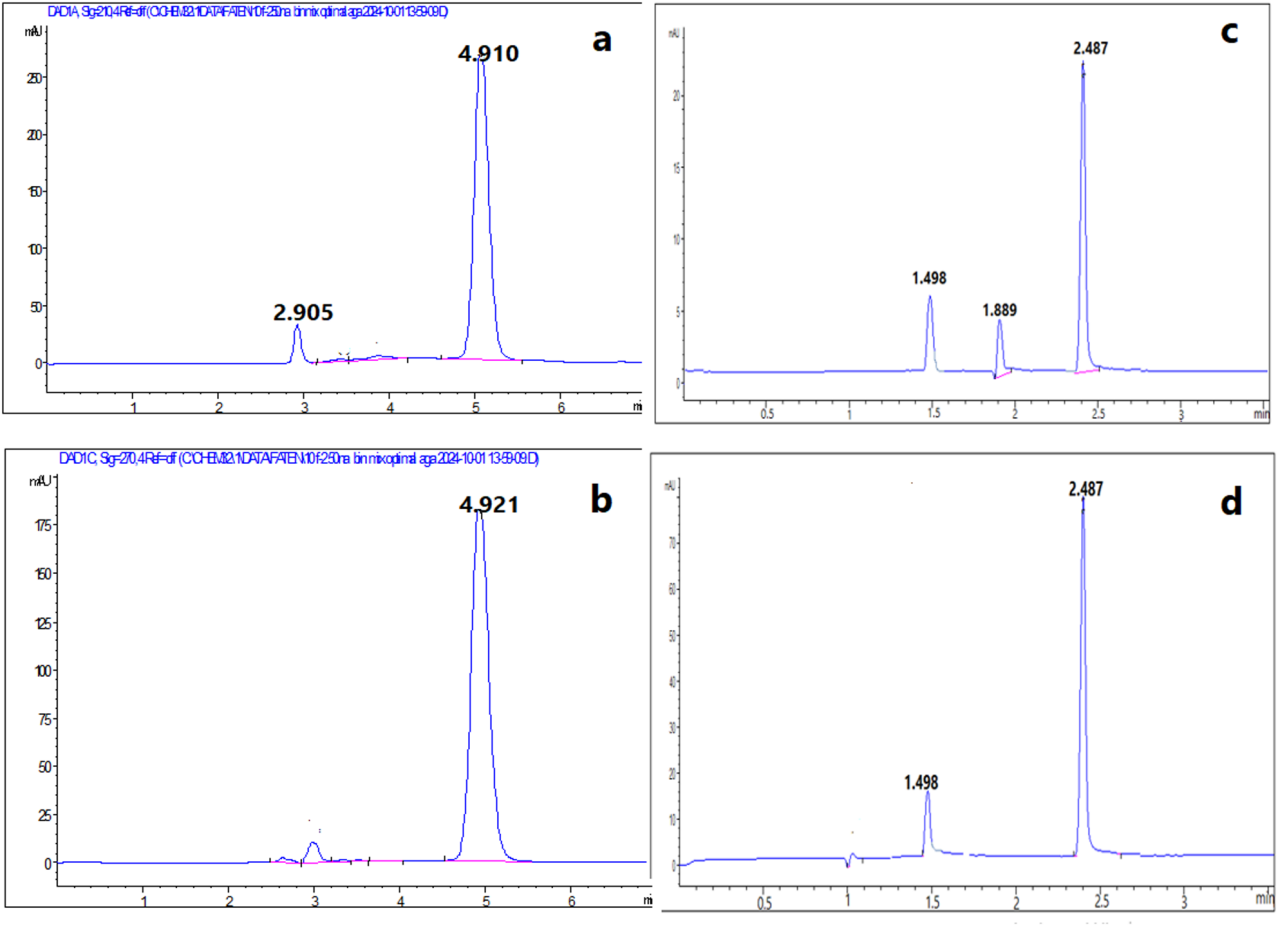
Laboratory-prepared eye drops containing mixture of 250 µg/mL NAT, 10 µg/mL FLC and 1 µg/mL BNZ using RP-HPLC **a** at 210 nm and **b** at 270 nm, as well as containing mixture of 1000 NAT µg/mL, 40 µg/mL FLC and 4 µg/mL BNZ using CZE **c** at 210 nm and **d** at 305 nm

The proposed methods were applicable for determination of combination of NAT and FLC in its laboratory-prepared eye drops with accepted %recoveries and %RSD. It was observed the absence of BNZ peak (preservative) in HPLC chromatograms which ensured the high selectivity of the proposed RP-HPLC method for simultaneous analysis of NAT and FLC. But BNZ peak has been separated in electropherograms at 1.49 min without any interference with the investigated drugs as shown in Fig. 6. So, each drug has been determined at its maximum wavelength with high selectivity. The statistical results of the proposed methods compared with the reported ones where FLC results were compared to results of (Wallace et al. 1992) [24] and NAT results were compared with results of (Paseiro-Cerrato, R., et al., 2013) [25] (Table 5). The Student’s t-test and F-test showed the high degree of agreement of the proposed methods with the reported ones.

### Analysis of pharmaceutical preparation

**Fig. 7:**
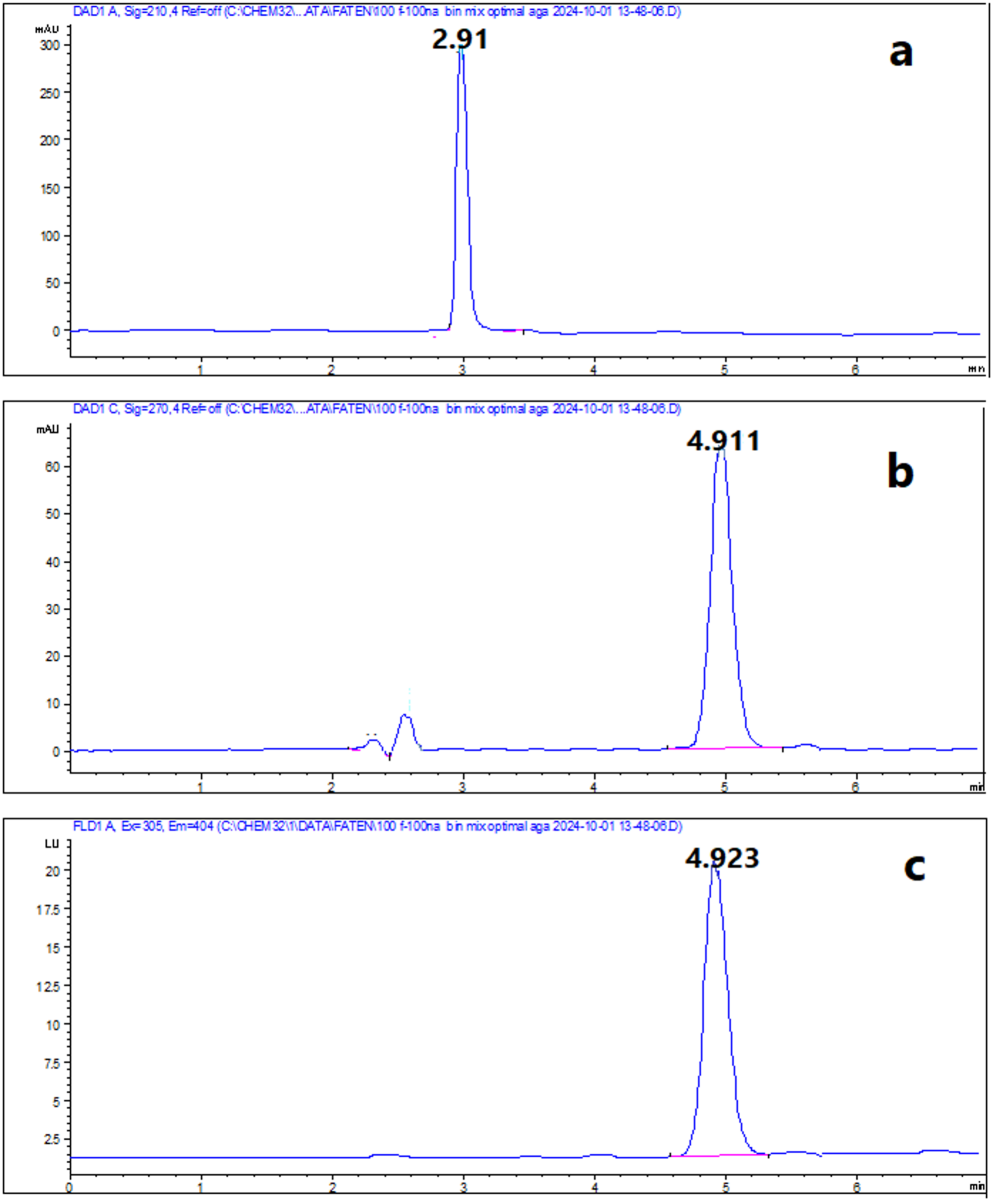
Typical chromatograms of pharmaceutical preparations of **a**100 µg/mL Diflucan^®^ IV solution at 210 nm, **b**100 µg/mL Natamycin^®^ eye drop at 270 nm and **c** FLD

**Fig. 8:**
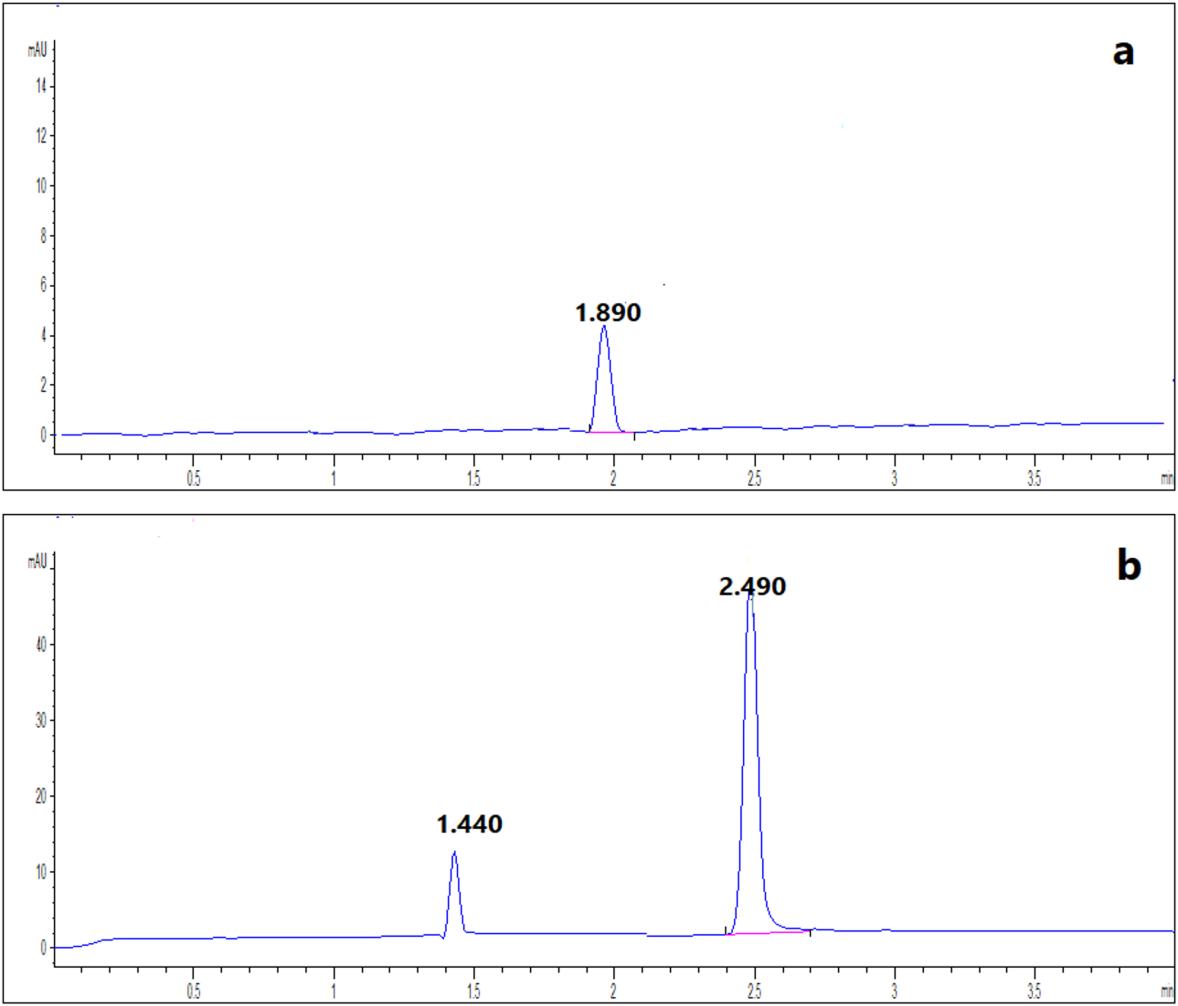
Typical electropherograms of pharmaceutical preparations of **a**100 µg/mL Diflucan^®^ IV solution at 210 nm and **b** 500 µg/mL Natamycin^®^ eye drop at 305 nm showing BNZ preservative at 1.44 min

Accuracy and precision of the proposed methods were ensured by obtained results of %Recovery and %RSD (Table 5). This ensured that the proposed methods were applicable for assay of investigated drugs in their pharmaceutical dosage form (Figs. 7 and 8).

**Table 5:** Application of the proposed methods for simultaneous determination of NAT and FLC in laboratory-prepared eye drop

Pharmaceutical preparation	Average %recovery ± %RSD^a^					
	RP-HPLC method		CZE method		Reported HPLC (FLC) [24]	Reported HPLC (NAT) [25]
Laboratory- prepared eye drop	FLC	NAT	FLC	NAT	FLC	NAT
	99.82 ± 1.38	100.56 ± 1.00	100.36 ± 1.59	99.88 ± 1.25	100.69 ± 1.69	101.12 ± 0.15
Natamycin^®^	100.72 ± 0.45	101.46 ± 0.96	100.57 ± 1.25	98.14 ± 1.07		
Diflucan^®^	101.63 ± 1.10	98.68 ± 1.05	99.25 ± 0.95	100.65 ± 0.74		
t^b^	1.05 (2.36)	0.78 (2.41)	1.50 (2.22)	1.67 (2.69)		
F^c^	2.56 (3.69)	1.99 (4.25)	1.97 (6.25)	2.32 (4.61)		

^a^ Average of percentage recoveries ± Relative standard deviation of five determinations

^b^ Figures between parentheses represent the corresponding tabulated values of t at *P* = 0.05

^c^ Figures between parentheses represent the corresponding tabulated values of F at *P* = 0.05

### Comparative analysis of greenness, whiteness and blueness tools

In this work, a variety of techniques for evaluating greenness have been outlined based on their criteria and methodologies. These different parameters involved in the use of each evaluation tool, along with their benefits and drawbacks, to create a comparative analysis of the assessment methods studied [12]. The NEMI tool is unique with its simplicity; however, when compared to other tools, it provides significantly limited and primarily qualitative data [11]. Furthermore, the modified NEMI, developed by Raynie & Driver, offers more comprehensive insights and a deeper understanding of each of the five evaluation criteria than NEMI approach, particularly by including energy consumption. Modified NEMI depends on three color levels involving green, yellow and red. The analytical Eco-scale offers a credible assessment of greenness in terms of accuracy and consistency. It effectively utilizes numerical values to reference greenness, facilitating comparisons between different analytical methods [13]. However, its primary limitation lies in its failure to provide comprehensive justifications for processes that are not green. Furthermore, the HPLC-EAT focuses mainly on the solvents that are consumed and produced during chromatographic techniques, neglecting other important factors such as energy usage, sample management, and instrumentation. In contrast, the GAPI tool takes into account all stages involved in the execution of an analytical procedure, from sample collection to the final outcome [16]. The most effective methods for assessing the greenness of analytical procedures are GAPI and AGREE, both of which deliver integrated insights regarding the entire applicable methodology. AGREE offers benefits such as simplicity, automation, and the ability to compute numerical values, which are advantages over GAPI [15]. It is recognized as the most effective method for adhering to GAC’s 12 principles. In conclusion, it is advisable to employ multiple greenness evaluation tools to achieve more trustworthy and reliable results, particularly when utilizing Eco-Scale, GAPI, and AGREE. Moreover, HPLC-EAT is a greenness assessment approach which evaluates the sustainability of HPLC methods by calculation of environmental, safety and health hazards of different solvents in chromatographic methods [26]. Another extension of GAC is white analytical Chemistry which involves three colors with a score, red to ensure analytical efficiency, green to ensures environmental effect and blue to indicate economic and practical aspects. For RGB score, a score of 50 indicates unsatisfactory method, a score of 75 indicates quit good results, but a score of 100 for highly applicable, economic, efficient and eco -friendly method [19]. Evaluation of methods practicality was performed using blue applicability grade index (BAGI) which is a complementary tool to green metrics. Assessment of BAGI results involves color and score value from 25 to 100. The from 75 to 100 indicates high applicability of the analytical method. VIGI is an innovative metric based on three scores (0, 5 and 10) which represents white, light violet and dark violet, respectively. VIGI interpretation resembles that of AGREE and BAGI as it depends on two key parameters. First, the intensity of color indicates the level of innovation: the darker the violet, the more innovative the analytical method. Second, the number in the inner part indicates the overall score of the analytical method (0-100). The higher ensures more innovative method [20].

## National environmental method index (NEMI)

**Fig. 9:**
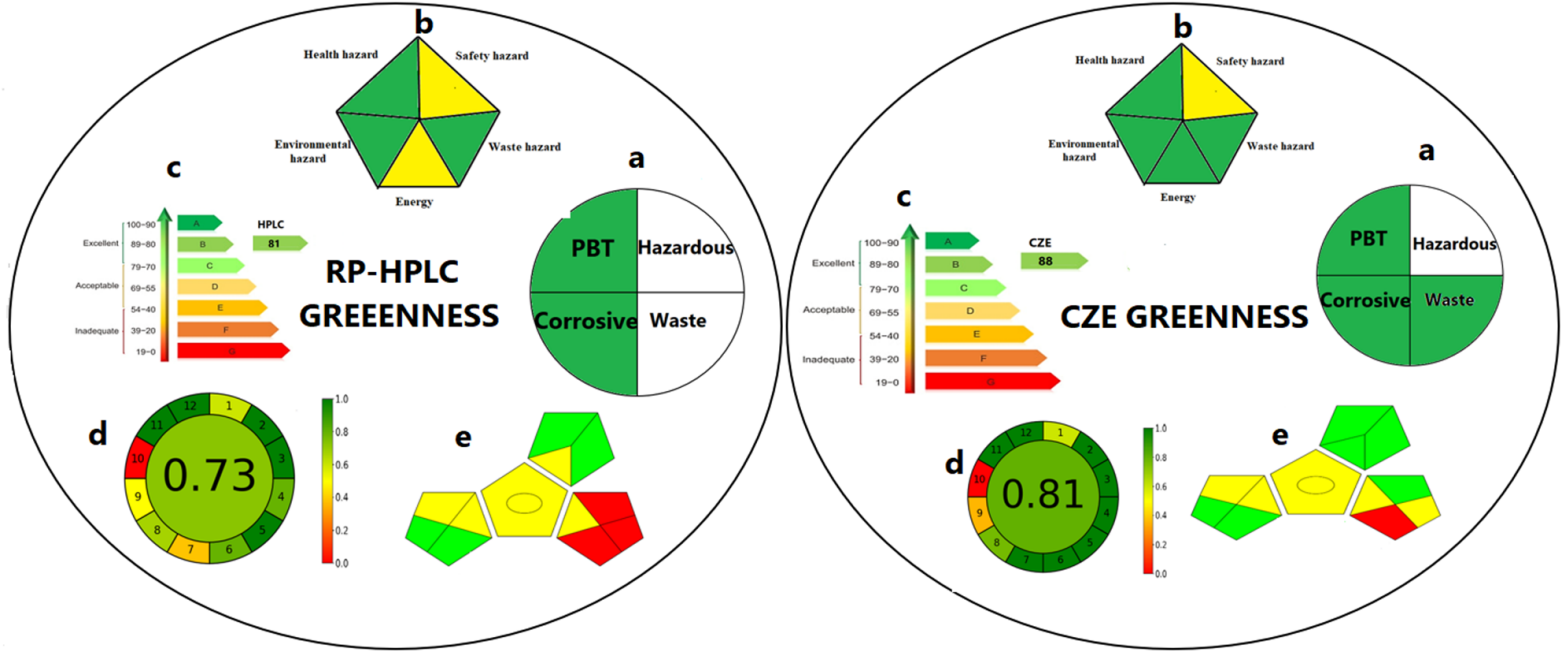
Greenness assessment of RP-HPLC and CZE methods using different greenness approaches

The first applied approach was national environmental method index (NEMI). It evaluates the analytical methods according to four aspects involving persistent, bio accumulative-toxic (PBT), corrosive, hazardous of waste and amount of waste generated within the procedure [11]. As shown in Fig. 9a, CZE method is more ecofriendly according to NEMI as RP-HPLC method produced larger amount of waste than CZE method. The two proposed methods have advantages of containing none of the chemicals in PBT list or corrosive chemicals with pHs within 2–12 range. This greenness assessment tool has an advantage as it is so easy to understand by researchers.

## Modified NEMI (Raynie and driver approach)

It is a modified form of NEMI tool which involves five aspects involving energy, environment, safety, health and amount of waste. This tool forms of pentagon pictograms with green, yellow or red color scale [12]. It is a modified form of NEMI as it contains additional parameter (energy consumption) and it is considered a semi-quantitative approach. In Fig. 9b, the CZE method is more advantageous than the RP-HPLC one in consuming less energy.

## Analytical eco-scale (AES)

AES is greenness assessment tool which depends on calculation of penalty points according to the used solvent and reagents types, solvents amount, waste consumption and energy consumption (Supplementary table 1a). The analytical method is considered to be excellent if analytical eco-scale is more than 75 [13]. As shown in Fig. 9c, RP-HPLC and CZE methods have 81 and 88, respectively. Green certificate modified eco-scale (GCES) is a new version of AES, it is easier than AES as it has diagrammatic representation.

## Analytical greenness metric (AGREE)

AGREE is a new tool for evaluation of greenness depending on twelve principles. AGREE can be performed using free online software which produces a pictogram with a score that varies from 0 to 1 [15]. The proposed methods have AGREE score larger than 0.6 which means that they are found in green region (Fig. 9d). Yet, it was found that CZE method has AGREE value larger than that of the RP-HPLC method.

## Green analytical procedure index (GAPI)

GAPI is a quantitative tool for assessment of greenness which involves many pictograms to demonstrate analytical procedure from sample preparation to the end of the analytical procedure [16]. It has three-color scale: green, yellow and red color, where green color indicates that the method is highly eco-friendly (Supplementary table 1b). RP-HPLC method consumed larger amount of solvent and produced larger amount of waste than CZE. So, GAPI pictograms of CZE method has green regions more than RP-HPLC method (Fig. 9e).

## HPLC environmental assessment (HPLC-EAT)

HPLC-EAT is a greenness assessment approach which evaluates the sustainability of HPLC methods by calculation of environmental, safety and health hazards [17]. The lower the EAT value the greener the analytical method. The score of the method was obtained from the following Eq. 

1 $$ \begin{aligned} {\mathrm{Score}}{\mkern 1mu} \, = & {\mkern 1mu} \,{\mathrm{S}}_{{\mathrm{1}}} {\mathrm{m}}_{{\mathrm{1}}} {\mkern 1mu} + {\mkern 1mu} {\mathrm{H}}_{{\mathrm{1}}} {\mathrm{m}}_{{\mathrm{1}}} + {\mathrm{E}}_{{\mathrm{1}}} {\mathrm{m}}_{{\mathrm{1}}} + {\mathrm{S}}_{{\mathrm{2}}} {\mathrm{m}}_{{\mathrm{2}}} + {\mathrm{H}}_{{\mathrm{2}}} {\mathrm{m}}_{{\mathrm{2}}} \\ & + {\mathrm{E}}_{{\mathrm{2}}} {\mathrm{m}}_{{\mathrm{2}}} + {\text{ }} \ldots {\text{ }} \ldots + {\mathrm{S}}_{{\mathrm{n}}} {\mathrm{m}}_{{\mathrm{n}}} + {\text{ H}}_{{\mathrm{n}}} {\mathrm{m}}_{{\mathrm{n}}} + {\text{ E}}_{{\mathrm{n}}} {\mathrm{m}}_{{\mathrm{n}}} \\ \end{aligned} $$

**Fig. 10:**
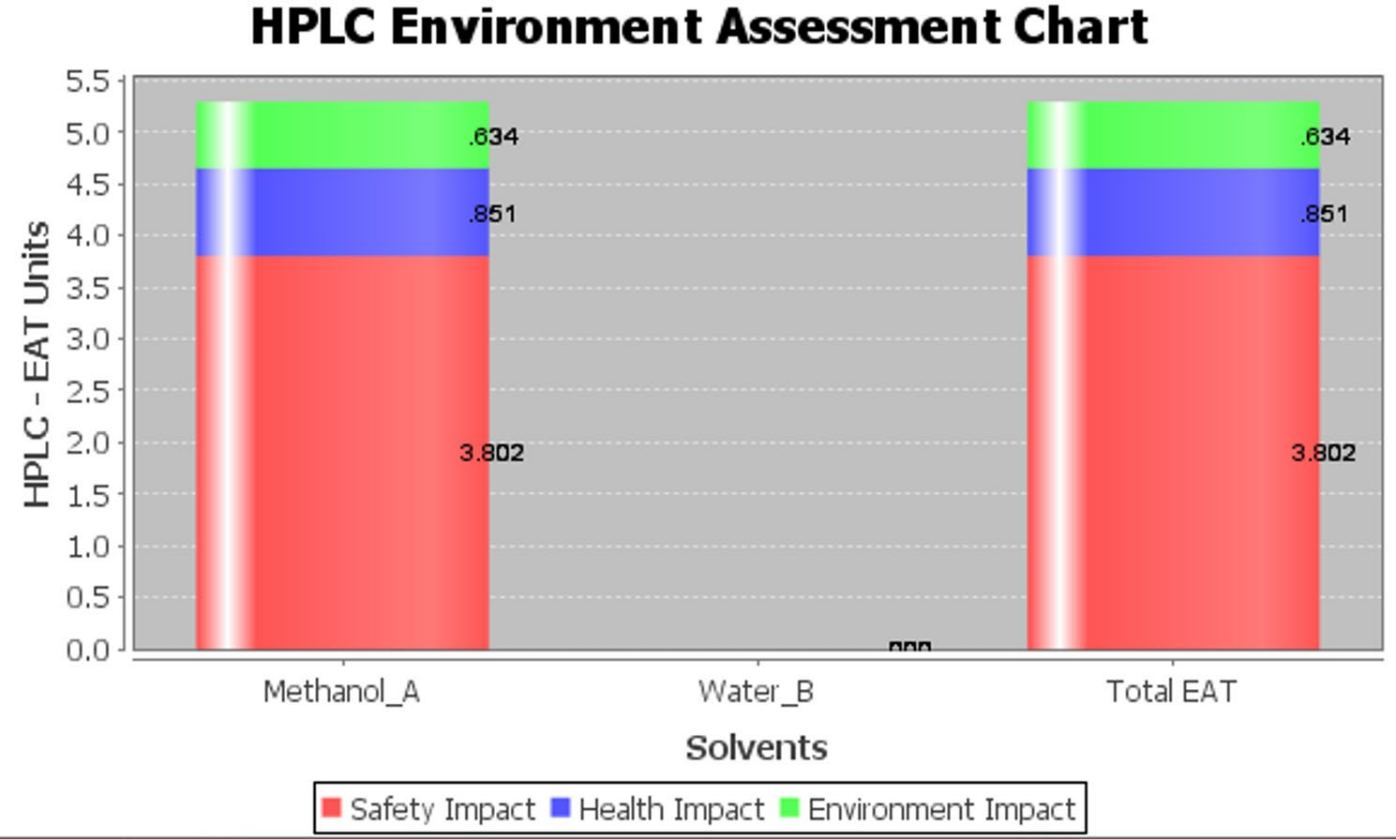
HPLC-EAT approach for RP-HPLC method

This equation defines H as health, S as safety, and E as the environment; m is the mass of the solvents, and n represents the number of solvents. Using software is another method to obtain HPLC-EAT score to facilitate the calculation [26]. Figure 10 shows that the proposed RP-HPLC was eco-friendly with small EAT values due to using of water in mobile phase composition.

## Whiteness assessment (RGB)

**Fig. 11:**
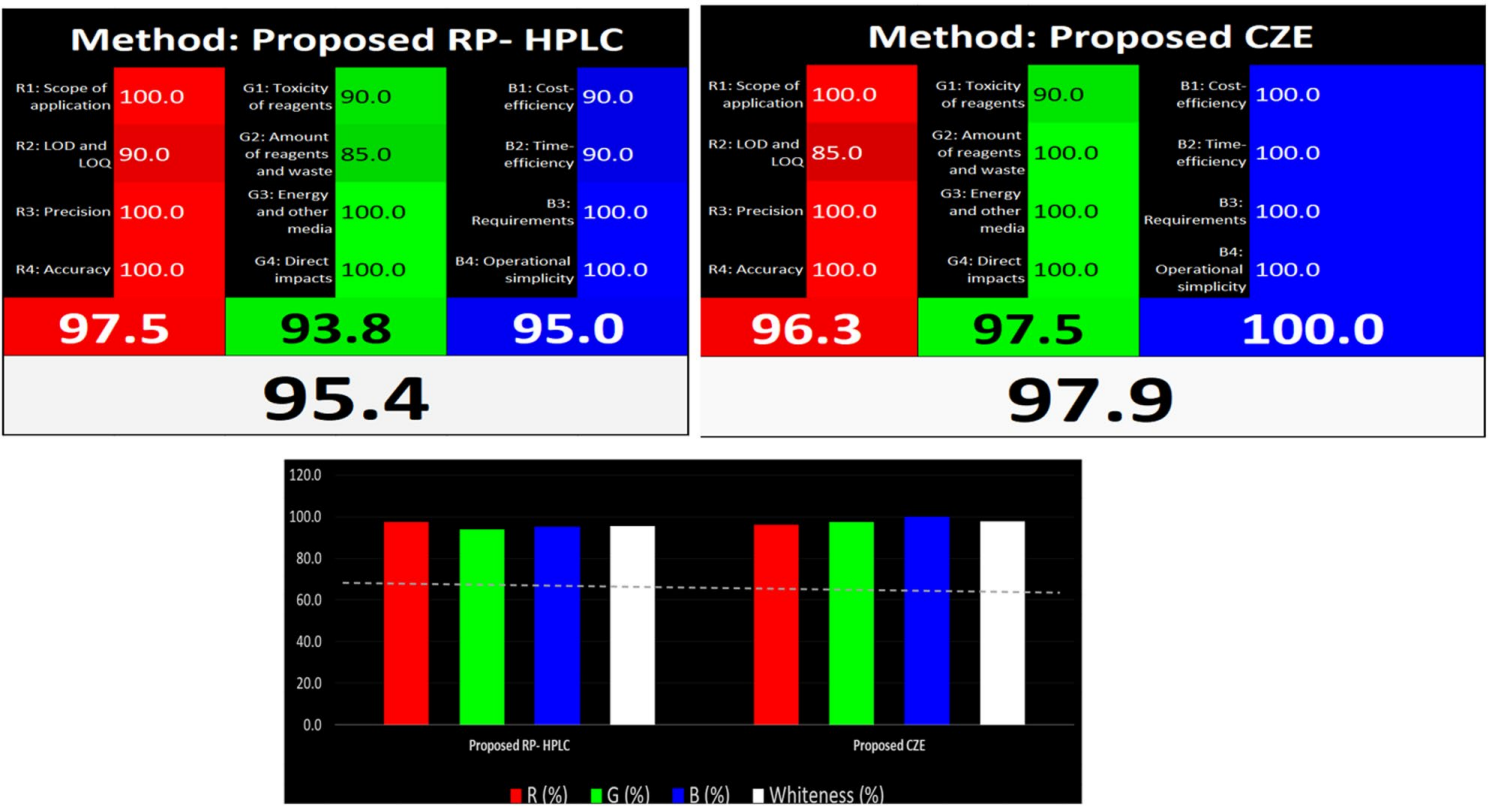
Whiteness pictograms using 12 RGB principles

As demonstrated in Fig. 11, RP-HPLC method has a red value larger than CZE method being more sensitive. This indicates the high efficiency of the RP-HPLC. On the other hand, CZE method has larger green and blue values than RP-HPLC method; this is because of the high cost of HPLC technique, consuming a huge amount of solvents and reagents. Overall, CZE method was more practical and economic than RP-HPLC method.

### Blueness assessment

**Fig. 12:**
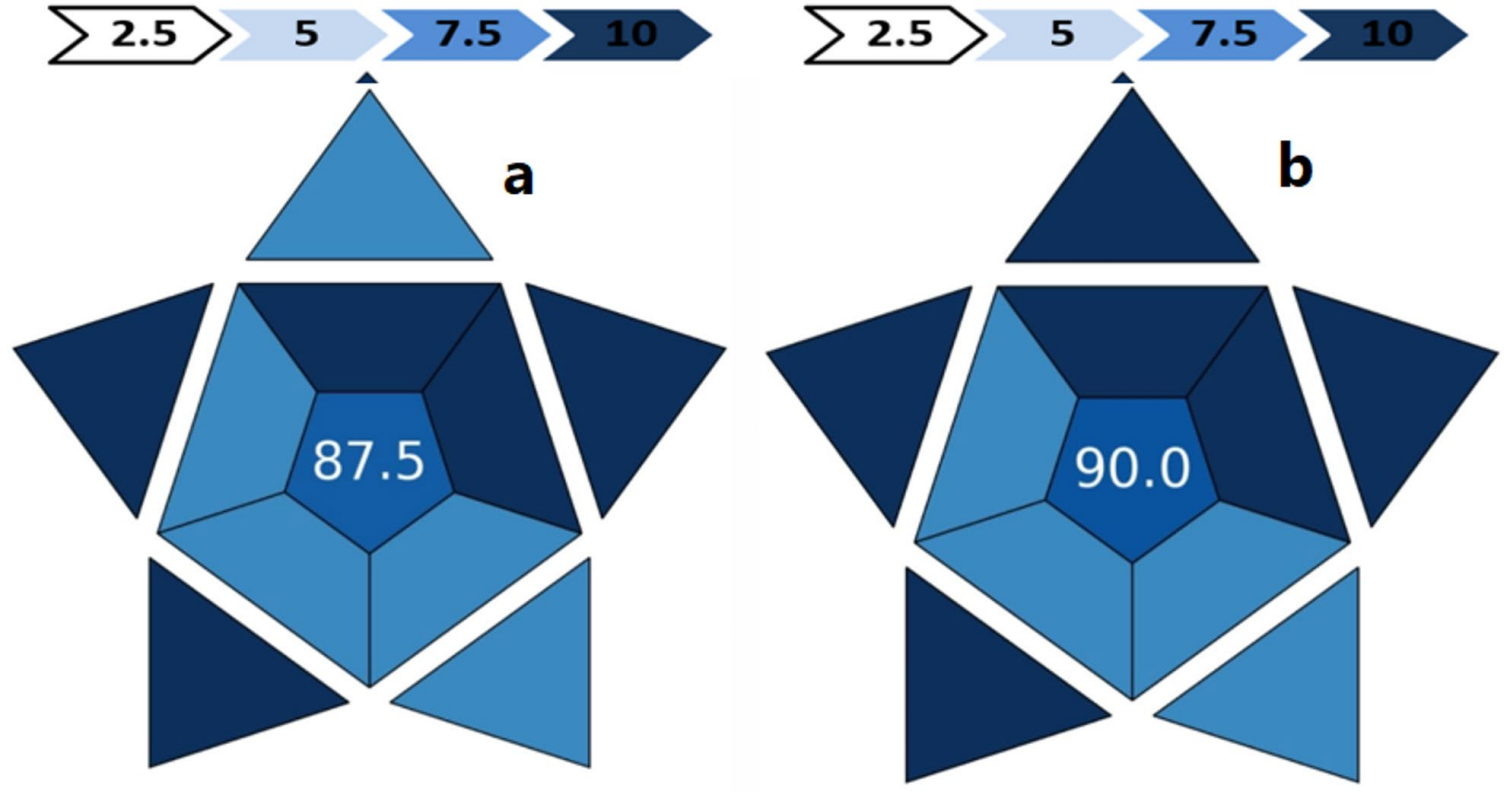
BAGI assessment for HPLC (**a**) and CZE (**b**)

Evaluation of methods practicality was performed using blue applicability grade indfex (BAGI) which is a complementary tool to green metrics. BAGI tool depends on evaluation of ten main parameters involving number of investigated drugs, number of measured samples per hour, type of analytical method, type of analysis, preconcentration requirements, number of samples that are simultaneously determined, degree of automation, sample amount, sample preparation, the type of used reagents and solvents and type of preparation of analytes. There are four different colors with different grades including white for no compliance, light blue for low compliance, blue for medium compliance and dark blue for the highest compliance [20]. Pictograms ensured the applicability of the two proposed methods which have high central values far from 25 and around 100. It was found that CZE method (score is 90) was more applicable than RP-HPLC method (score is 87.5) one (Fig. 12). This small difference in the applicability value between the proposed methods was due to the ability of CZE method to analyze a larger number of samples per hour than RP-HPLC method (Supplementary Table 2).

### Violet innovation grade index (VIGI)

**Fig. 13:**
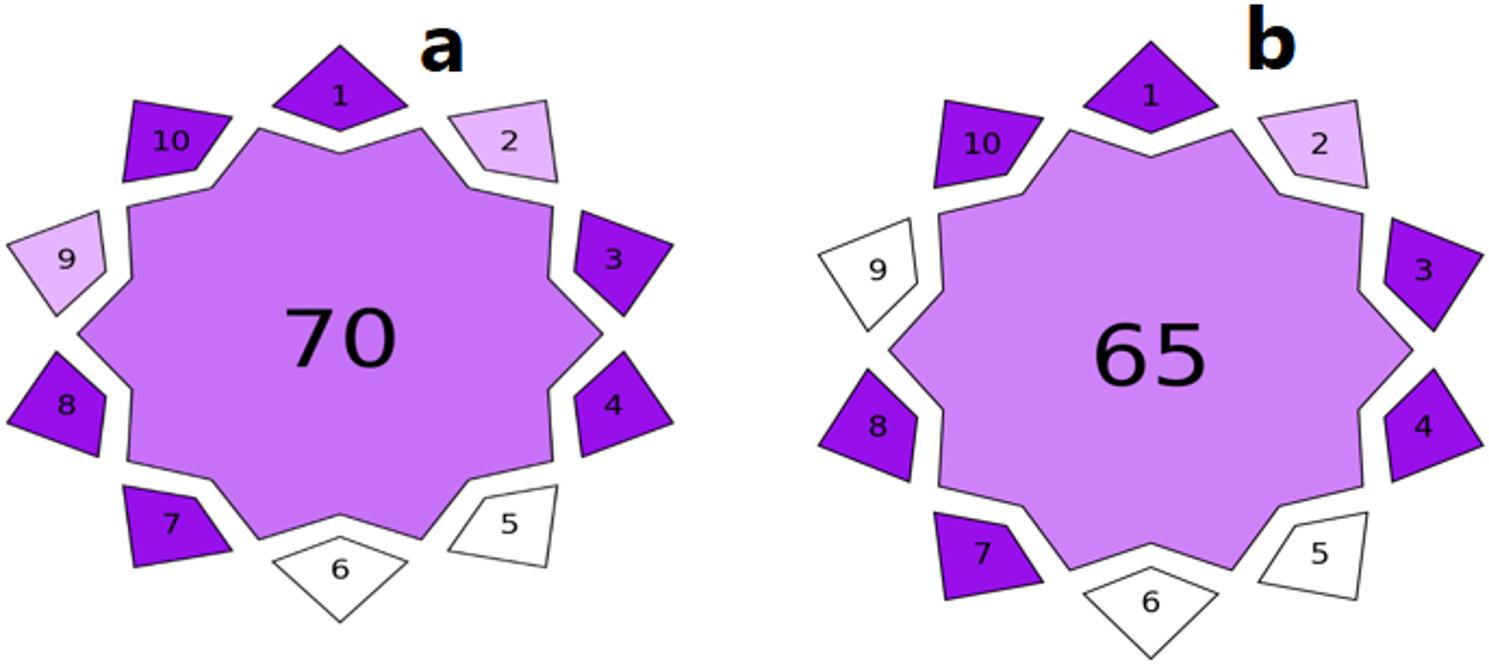
VIGI pictograms for RP-HPLC method (**a**) and CZE method (**b**)

After application of main features and criteria of VIGI tool it was ensures that the proposed methods are highly innovative. However, the RP-HPLC method is more innovative than CZE method as it has a higher sensitivity (Fig. 13). The pentagon pictogram was obtained by using software after downloading it using the link https://bit.ly/VIGItool.

## Conclusion

The proposed methods are the first work on simultaneous analysis of NAT and FLC combination either in raw materials or in ophthalmic preparations. The proposed RP-HPLC was developed as the primary, high-sensitivity workhorse method, ideal for routine quality control in well-equipped laboratories where maximum sensitivity for NAT (especially with FLD) is paramount. While the CZE method has superior sustainability and is more cost-effectiveness offering high-throughput screening and orthogonal verification to HPLC where it operates on a completely different separation mechanism (charge-to-size ratio vs. hydrophobicity in HPLC). It serves as an orthogonal method to confirm HPLC results in case of ambiguous peaks or matrix interference, a key principle in analytical quality-by-design. They have the advantage of overcoming the problem of the high ratio between NAT and FLC in ophthalmic preparation (25:1). Greenness, whiteness and blueness assessments ensure the sustainability, efficiency and applicability of the proposed methods for routine analysis of the investigated drugs as an essential quality control tool for effective clinical studies.

## Supplementary Information

Below is the link to the electronic supplementary material.

Supplementary Material 1

## Data Availability

The datasets generated or analysed during this study are available from the corresponding author on reasonable request.

## Abbreviations

NAT: Natamycin
FLC: Fluconazole
BNZ: Benzalkonium chloride
DAD: Diode array detector
FLD: Fluorescence detector
RP-HPLC: Reversed phase-high-performance liquid chromatographic method
CZE: Capillary zone electrophoresis
S/N: Signal to noise ratio
ICH: International conference on harmonization
S_a_: Standard deviations of the intercept
S_b_: Standard deviations of the Slope
r: Correlation coefficient
S_y/x_: Standard deviations of residuals
WAC: White analytical chemistry
NEMI: National environmental method index
AGREE: Analytical Greenness Metric
GAPI: Green Analytical Procedure Index
RGB: Red- Green- Blue method
BAGI: Blue Applicability Grade Index
VIGI: Violet Innovation Grade Index
SD: Sustainable development
LOD: Limit of detection
LOQ: Limit of quantification
SD: Standard deviation
R%: Percentage recovery
RSD%: Percentage relative standard deviation
Er%: Percentage relative error
SSP: System suitability parameters
t_R_: Retention time
k': Retention factor
N: Number of theoretical plates
ɑ: Selectivity factor
