# Supplementary material for: Sustainable chromatographic assays of a novel antifungal combination for keratomycosis
Source: BMC Chem. 2026 Mar 16;20(1):76. doi: 10.1186/s13065-026-01735-y (PMC13063551; doi:10.1186/s13065-026-01735-y)
Supplement: Supplementary file 1 — Supplementary Material 1 [file 13065_2026_1735_MOESM1_ESM.docx]

| **Supplementary table 1: The penalty points (a) and Green Analytical Procedure index (b) for the analysis of laboratory-prepared mixture of FLC and NAT in pharmaceutical preparation.** | | | | |
| --- | --- | --- | --- | --- |
| **(a) Analytical Eco-scale** | | | | |
| **Reagents/instruments** | **Penalty points** | | | |
|  | **HPLC Method** | | **CZE Method** | |
| Methanol | 12 | | 6 | |
| Borate buffer | -- | | 2 | |
| Distilled water | Not hazardous | | Not hazardous | |
| HPLC | 2 | | -- | |
| CE-DAD | -- | | 1 | |
| Occupational hazard | 0 | | 0 | |
| Waste >10mL | 5 | | 3 | |
| Total penalty points | 19 | | 12 | |
| Analytical Eco-Scale total score | 81 | | 88 | |
| **(b) Green Analytical Procedure Index** | | | | |
| **Reagents/instruments** | | **HPLC Method** | | **CZE Method** |
| **Sample Preparation** | | | | |
| Collection (1) | | at-line | at-line | |
| Preservation (2) | | None | None | |
| Transport (3) | | None | None | |
| Storage (4) | | Under normal conditions | Under normal conditions | |
| Type of method: direct or indirect (5) | | Filtration and degasing | Filtration | |
| Scale of extraction (6) | | -- | -- | |
| Solvents/reagents used (7) | | -- | -- | |
| Additional treatments (8) | | -- | -- | |
| **Reagent and solvents** | | | | |
| Amount (9) | | 10-100 mL | <10 mL | |
| Health hazard (10) | | NFPA health hazard score= 1 | NFPA health hazard score= 1 | |
| Safety hazard (11) | | instability score =0 | instability score =0 | |
| **Instrumentation** | | | | |
| Energy (12) | | ≤1.5 kWh per sample | ≤1.5 kWh per sample | |
| Occupational hazard (13) | | **-** | Hermetic sealing of analytical method | |
| Waste (14) | | 10 mL< | 1-10mL | |
| Waste treatment (15) | | No treatment | No treatment | |
| Quantification | | Yes | Yes | |

| **Supplementary table 2: BAGI assessment of the proposed methods** | | |
| --- | --- | --- |
| **Parameters** | **RP-HPLC Method** | **CZE Method** |
| Type of analysis | Quantitative and confirmatory | Quantitative and confirmatory |
| Number of investigated samples | 2-15 compounds of different chemical classes | 2-15 compounds of different chemical classes |
| Analytical technique | Simple instrumentation (HPLC-DAD) | Simple instrumentation (CZE-DAD) |
| Simultaneous sample preparation | 13-95 | 13-95 |
| Sample preparation | Not required | Not required |
| Sample /h (sample preparation+ analysis time) | 5-10 | 10< |
| Reagents and materials | Common commercially available reagents (methanol) | Common commercially available reagents (buffer) |
| Preconcentration | No preconcentration is required | No preconcentration is required |
| Automation degree | Semi-automated | Semi-automated |
| Amount of sample | 10 mL> | 10 mL> |
